# Evaluation of* In Vitro* Antioxidant and Antidiabetic Activities of* Aristolochia longa* Extracts

**DOI:** 10.1155/2019/7384735

**Published:** 2019-04-02

**Authors:** Nasreddine El Omari, Karima Sayah, Saad Fettach, Omar El Blidi, Abdelhakim Bouyahya, My El Abbes Faouzi, Rabie Kamal, Malika Barkiyou

**Affiliations:** ^1^Laboratory of Histology, Embryology and Cytogenetic, Faculty of Medicine and Pharmacy, Mohammed V University of Rabat, Morocco; ^2^Biopharmaceutical and Toxicological Analysis Research Team, Laboratory of Pharmacology and Toxicology, Faculty of Medicine and Pharmacy, Mohammed V University of Rabat, Morocco; ^3^Laboratory of Human Pathology Biology, Faculty of Sciences, Genomic Center of Human Pathology, Faculty of Medicine and Pharmacy, Mohammed V University of Rabat, Morocco

## Abstract

Oxidative stress plays a major role in diabetic physiopathology; hence, the interest of using natural antioxidants as therapeutic tools exists. The aim of this study was the evaluation of* in vitro* antioxidant activity and inhibitory potential of organic extracts from* Aristolochia longa* roots against key enzymes linked to hyperglycemia. Antioxidant activity was performed using 2,2′-diphenyl-1-picrylhydrazyl (DPPH) and 2,2-azino-bis-3-ethylbenzothiazoline-6-sulfonic acid (ABTS) radicals and ferric reducing/antioxidant power (FRAP) methods. The *α*-Glucosidase and *β*-Galactosidase inhibitory activities were investigated using an* in vitro* model. Moreover, phytochemical analysis of tested extracts was carried out. The aqueous fraction of this herb exhibited the highest antioxidant activity for both DPPH and ABTS methods, IC_50_=125.40±2.40 *μ*g/mL and IC_50_=65.23±2.49 *μ*g/mL, respectively. However, the ethyl acetate fraction possessed the strongest inhibitory effect towards *α*-Glucosidase (IC_50_=1.112±0.026 mg/mL). Furthermore, the result showed high levels of phenolic content. The results showed that this plant could be a significant source of medically important natural compounds.

## 1. Introduction

Medicinal plants are one of the main resources of therapeutic agents. Indeed, 80% of the world's population uses plants in health care [1]. Recently, the interest in the search for natural substances has considerably increased, because these substances are intended for use in foods or drugs to replace synthetic compounds, which are limited because of their side effects [2]. There is an increasing interest in using medicinal plants and their phytoconstituents as natural sources because of their well-known ability to scavenge free radicals. Effectively, plants are sources of natural antioxidants compounds that possess various pharmacological properties with little or no side effects and protect human health from many diseases [3–5]. The prevention of oxidative stress related disease by medicinal plant products is delaying the oxidation of lipids or other molecules by inhibiting the propagation of oxidative chain reactions [2].

Among Moroccan medicinal plants,* Aristolochia longa* (*A. longa*) is a medicinal plant belonging to Aristolochiaceae family, which is widely distributed in the tropical and temperate regions [6]. Aristolochia species contain secondary metabolites that have well-known beneficial effects [7].* A. longa* locally known as “Barraztam” is a species commonly used in Moroccan traditional medicine. Many traditional healers also use a small amount of its rhizome powder with honey or salted butter for the treatment of abdominal pain and upper respiratory tract infections [8–10].

Likewise, diabetes mellitus is a major cause of mortality and the most common metabolic disorder characterized by hyperglycemia due to lack of insulin production by the pancreas or the inability of the insulin produced to control blood glucose [11, 12]. One interesting approach is to reduce postprandial hyperglycemia by retarding glucose uptake through the inhibition of carbohydrate-hydrolyzing enzymes, such as *α*-Glucosidase and *β*-Galactosidase [13, 14]. In this context, the aim of this study was to evaluate the* in vitro* antioxidant activity, *α*-Glucosidase and *β*-Galactosidase inhibitory potentials of* A. longa* root extracts.

## 2. Materials and Methods

### 2.1. Reagents

*p*-Nitrophenyl-*α*-D-glucopyranoside, 2-Nitrophenyl-*β*-D-galactopyranoside, *α*-Glucosidase from* Saccharomyces cerevisiae, β-*Galactosidase from* Aspergillus oryzae*, Acarbose, Folin-Ciocalteu reagent, rutin, catechin, 2,2′-diphenyl-1-picrylhydrazyl (DPPH), 2,2-azino-bis-3-ethylbenzothiazoline-6-sulfonic acid (ABTS), 6-hydroxy-2,5,7,8-tetramethylchroman-2-carboxylic acid (Trolox), and ascorbic acid were purchased from Sigma-Aldrich (France). All other reagents were of analytical grade.

### 2.2. Plant Material

#### 2.2.1. Plant Collection

Roots of* A. longa* were collected in April 2016, in the province of Al Haouz in Morocco. The collected plant materials were authenticated at the Herbarium of Botany Department of the Scientific Institute of Rabat, Morocco. The roots of the plant were washed, dried at room temperature from 48 to 92 h, grounded into powder, and then stored in glass bottles preserved from light and moisture until use.

#### 2.2.2. Preparation of Plant Extracts

To prepare the extracts, the technique of continuous hot extraction by a Soxhlet extractor was carried out using solvents of different polarities. Briefly, 10 g of root powder was extracted successively with 100 mL each of ethyl acetate, methanol, and water until the extracts were colorless in the siphon tube. The aqueous extract was prepared by adding 500 mL of distilled water to 50 g of* A. longa* dry roots powder. After 24 h of maceration under magnetic stirring at room temperature, the mixture was centrifuged. Then, all extracts already prepared were filtered through a filter paper (Whatman) and evaporated to dryness by a rotary evaporator at 50°C. The extracts obtained were kept at 4°C until further uses.

### 2.3. Phytochemical Analysis

#### 2.3.1. Determination of Total Phenolic Content

Total phenolic content of* A. longa* extracts was assessed by Folin-Ciocalteu method [15] as described by Spanos and Wrolstad [16]. Gallic acid was used as standard for the calibration curve and the results are expressed as mg of gallic acid equivalent per g of extract dry weight (mg GAE/g edw).

#### 2.3.2. Determination of Total Flavonoid Content

The total flavonoid content of* A. longa* extracts was determined according to the method described by Dewanto et al. [17] using Aluminium Chloride (AlCl3). Rutin was used to perform the standard curve, and the results were expressed as rutin equivalent per gram of extract dry weight (mg RE/g edw).

#### 2.3.3. Determination of Proanthocyanidin Content

The proanthocyanidin content of* A. longa* extracts was determined as reported by Julkunen-Tiitto [18]. Catechin was used as a standard for constructing the calibration curve and the results are expressed as catechin equivalent per gram of extract dry weight (mg CE/g edw).

### 2.4. Antioxidant Activity

#### 2.4.1. DPPH Radical Scavenging Activity Assay

Radical scavenging activity of the extracts was measured using the stable radical DPPH such as that determined by Sayah et al. [19] with some modifications. In tubes, 2.5 mL of different concentrations of each extract were introduced and 0.5 mL of methanol solution of DPPH (0.2 mM of DPPH, dissolved in methanol) freshly prepared was added. The mixture is vigorously vortexed and left in the dark at ambient temperature for 30 min. Then, the absorbance of the mixture was measured at 517 nm in a spectrophotometer. The antioxidant activity of our extracts is expressed as the percentage of DPPH radical inhibition and the IC_50_ was calculated for comparing the obtained results.

#### 2.4.2. ABTS Radical Scavenging Assay

The TEAC test (Trolox Equivalent Antioxidant Capacity) or discoloration test of ABTS***∙***^**+**^ was carried out according to the method described by Sayah et al. [19]. Briefly, the cationic radical (ABTS***∙***^**+**^) was prepared by the reaction between 10 mL of ABTS (2 mM) in H_2_O and 100 *μ*L of potassium persulfate (K2S2O8) (70 mM). The mixture was incubated in the dark for 16 hours at room temperature. Then, the ABTS***∙***^**+**^ solution was diluted with methanol to obtain an absorbance of 0.70 at 734 nm. Then, 200 *μ*L of each extract was mixed with 2 mL of the diluted ABTS***∙***^**+**^ solution and allowed to react for 1 minute. After, the absorbance of the ABTS***∙***^**+**^ radical is measured at 734 nm. All samples were made in triplicate. The results were represented as Trolox equivalent per gram of extract dry weight (mg TE/edw).

#### 2.4.3. Ferric Reducing/Antioxidant Power (FRAP) Assay

The FRAP test is performed according to the method described by Sayah et al. [19]. Briefly, 1 mL of each extract (1 mg/mL) was mixed with 2.5 mL of the phosphate buffer solution (0.2 M, pH 6.6) and 2.5 mL of the 1% potassium ferricyanide aqueous solution. After incubation at 50°C for 20 min, 2.5 mL of 10% trichloroacetic acid were added to the mixture, and then the mixture was centrifuged at 3000 rpm for 10 min. At the end, 2.5 mL of the supernatant was mixed with 2.5 mL of distilled water and 0.5 mL of aqueous ferric chloride solution FeCl_3_ (0.1%, w/v). The absorbance was measured at 700 nm. The results are expressed as ascorbic acid equivalent per gram of extract dry weight (mg AAE/g edw).

### 2.5. *In Vitro* Antidiabetic Effects

#### 2.5.1. *α*-Glucosidase Inhibitory Assay

The *α*-Glucosidase inhibitory activity was monitored using the substrate* p*-Nitrophenyl *α*-d-glucopyranoside (pNPG), which is hydrolyzed by *α*-Glucosidase to release p-Nitrophenyl (a colored agent which can be monitored at 405 nm), according to the method described by Marmouzi et al. [20, 21]. The results are expressed as percentage inhibition, while, for comparing results, the concentrations of inhibitor required to inhibit 50% of enzyme activity (IC_50_) were determined.

#### 2.5.2. *β*-Galactosidase Inhibitory Assay

The *β*-Galactosidase inhibitory activity was assessed according to the method of Bouabid et al. [22] using 2-Nitrophenyl *β*-D-Galactopyranoside as substrate, which is hydrolyzed by *β*-Galactosidase to release 2-nitrophenyl (a colored agent; which can be monitored at 410 nm). Briefly, a mixture of 150 *μ*L of the samples at different concentrations (0.5-5 mg/mL) and 100 *μ*L of sodium phosphate buffer 0.1 M (pH=7.6) containing the enzyme *β*-Galactosidase solution (0.1U/mL) was incubated at 37°C for 10 min. After preincubation, 200 *μ*L of gala solution 1 mM in sodium phosphate buffer 0.1 M (pH=7.6) was added. The reaction mixtures were incubated at 37°C for 30 min. After incubation, 1 mL of 0.1 M of Na_2_CO_3_ were added to stop the reaction and the absorbance was recorded at 405 nm using the spectrophotometer. The *β*-Galactosidase inhibitory activity was expressed as percentage inhibition and calculated using the same formula as the *α*-Glucosidase test, and the IC_50_ values were determined. Quercetin was used as positive control and the experiment was carried out in triplicate.

### 2.6. Statistical Analysis

Results are expressed as a mean ± standard error of the mean. Differences between the means were determined by one-way analysis of variance (one-way ANOVA). A difference in the mean values of *p* < 0.05 was considered to be statistically significant. All analyses were performed with GraphPad Prism 6. Also, IC_50_ values were determined using a nonlinear regression curve with the same program.

## 3. Results and Discussion

### 3.1. Total Phenolic, Flavonoid, and Proanthocyanidin Contents

Total phenolic, flavonoid, and proanthocyanidins contents are presented in Table 1. The phenolic contents in ethyl acetate fraction (EAF) of* A. longa* were found to be 32.55±0.43 mg GAE/g edw, which are significantly (*p*< 0.05) greater than methanolic fraction and aqueous extract (24.48±1.63 mg GAE/g edw and 13.13±0.48 mg GAE/g edw) respectively. There is no significant difference between EAF and aqueous fraction (AF) (28.90±1.36 mg GAE/g edw). Results of flavonoid and proanthocyanidin contents show that ethyl acetate fraction resulted also in significantly (*p*< 0.05) higher values of those compounds (116.86±4.12 mg RE/g edw and 77.64±1.93 mg CE/g edw, respectively) followed by the aqueous fractions that were 7.60±0.11 mg RE/g edw and 10.79±0.49 mg CE/g edw, respectively. The interesting phenolic contents of this herb indicate an important health promoting activity. Indeed, Djeridane et al. [23] worked on the* A. longa* roots and they estimated the total phenolics and flavonoids content as 1.47±0.02 mg GAE/g edw and 0.81±0.02 mg RE/g edw, respectively [23]. These compounds are secondary plant metabolites possessing a wide range of pharmacological activities such as anticancer, antiviral, anti-inflammatory activities and effects on capillary fragility [24–26]. Previous studies, which have been interested in other species of Aristolochia species such as* A. bracteolata* and* A. indica*, revealed that the methanol extract from each plant contains high amount of phenols and flavonoids [25, 26]. Phenolic compounds are an important and complex group of chemical constituents present in plants and are classic defense compounds to protect plants from herbivores, pathogenic, and parasite infections [27].

### 3.2. Antioxidant Activity

Recently, many scientific studies have shown that free radicals play a major role in the development of cancer, heart disease, aging, cataracts, and immune system damage [28]. These unstable free radicals can be eliminated by antioxidants that inhibit the rate of oxidation and protect cells from damage [28]. Antioxidant drugs are used for the prevention and treatment of oxidative stress related disease such as diabetes, Alzheimer's disease, atherosclerosis, stroke, and cancer [29, 30]. However, its side effects and high prices force many people to take herbal medicines, which have fewer side effects [31]. Numerous methods for the analysis of* in vitro* and* in vivo* antioxidant activity have been developed, but only a few fast and reliable methods for a large number of plant extract samples exist [32–34]. Furthermore, to study the antioxidant activity of our plant extracts, the ability to scavenge the stable free radical DPPH and the cation ABTS and their ferric reducing antioxidant power (FRAP) was evaluated.

**Figure 1:**
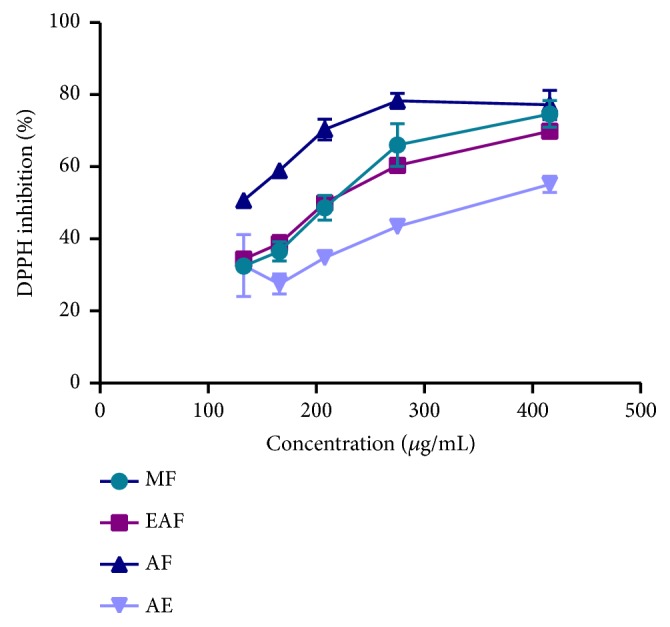
DPPH radical scavenging activity of extracts. The values are the mean of three determinations ± standard error.

DPPH is one of the stable free radicals, commercially available, nitrogen centered, and largely used for evaluating scavenging activity of antioxidant standards and plant extracts with a characteristic absorbance at 517 nm [35], which decreases in the presence of free radical scavengers. By accepting hydrogen from a corresponding donor, the DPPH solution loses the characteristic dark purple color and becomes yellow diphenylpicryl hydrazine [36–39]. This scavenging activity has been widely used as a quick and reliable parameter to evaluate the general* in vitro* antioxidant activity of plant extracts [40, 41]. Recently, numerous studies reported the antioxidant properties of medicinal plant products using DPPH assay [42–44]. From these assay several molecules from medicinal plants were developed as antioxidant agents.  Figure 1 illustrated the DPPH radical scavenging activity of different extracts of* A. longa* at various concentrations. All* A. longa* extracts showed scavenging effect, which increases with the concentration of samples. At 416 *μ*g/mL concentration, aqueous and methanolic fraction of* A. longa* exhibited increased DPPH radical scavenging activity of 77.17% and 74.66%, respectively. Moreover, aqueous extract showed less activity at all concentrations. These results showed that* A. longa* roots contained high amount of radical scavenging compounds with proton-donating ability. Similar result was observed in one previous study of Benmehdi et al. [45], which showed a dose-dependent scavenging of DPPH radicals using* A. clematitis* roots.

**Figure 2:**
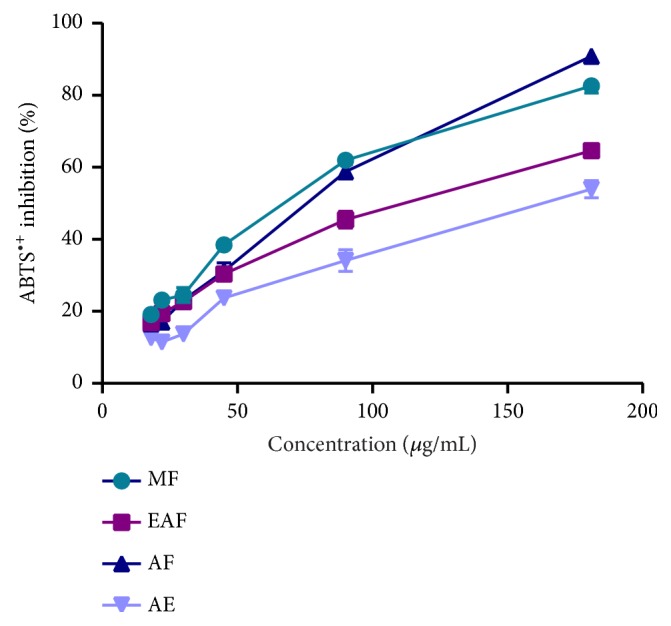
ABTS radical scavenging activity of extracts. The values are the mean of three determinations ± standard error.

The radical cation of 2,2-azino-bis-3-ethylbenzothiazoline-6-sulfonic acid (ABTS) is stable in its free form. The concentration of this radical can be determined by measuring the absorbance at 734 nm. The addition of an antioxidant to a solution of this radical leads to its reduction and a decrease in absorbance. This decrease depends on the antioxidant activity of the test compound, but also on the time and the concentration [46]. The experiments were carried out using an improved ABTS decolorization assay [47]. The ability of the extracts to scavenge ABTS cation was expressed in Figure 2. The aqueous fraction exhibited potent ABTS radical cation scavenging activity in concentration dependent manner. At 181 *μ*g/mL concentration, aqueous and methanolic fraction of* A. longa* possessed 90.89% and 82.58% scavenging activity on ABTS. At the concentrations investigated, this effect may indicate the capacity of the herb to minimize oxidative damage to certain vital tissues of the body [48]. These results are in agreement with the findings of Jegadeeswari et al. [49] using the same ABTS test on another Aristolochia species.

**Figure 3:**
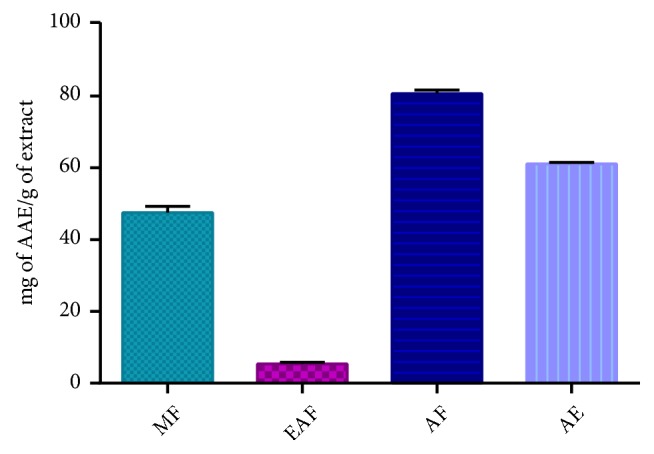
Ferric reducing antioxidant power of extracts. The values are the mean of three determinations ± standard error.

In the FRAP method, antioxidants present in the sample reduced the Fe3+/ferricyanide complex to the blue ferrous form [50], which can serve as a measure of the antioxidant capacity interpreted as the reducing power [51]. The reducing power of the extracts is represented in Figure 3. A higher absorbance indicates a higher reducing power. At 2000 *μ*g/mL concentration and among the solvents tested, aqueous fraction exhibited higher reducing activity 80.73%. The results obtained are consistent with the studies carried out on* A. longa* (aerial part) [52] and* A. indica *(aerial part) [53] and (roots) [54], which indicate that they have a reducing power.

To compare the results, the IC_50_ are calculated as shown in Table 2. A lower value of IC_50_ indicates greater antioxidant activity. Indeed, the aqueous fraction showed the highest DPPH radical inhibition value (IC_50_=125.40±2.40 *μ*g/mL), while the methanolic fraction was the most active against the ABTS radical cation (IC_50_=61.58±2.15 *μ*g/mL), and the Trolox value for both tests was 1.79±0.35 *μ*g/mL and 0.70±0.01 *μ*g/mL, respectively. Our results are in analogy to those of Ramalalingam et al. [53] using the DPPH assay on the aqueous extract of* A. indica*, who showed IC_50_ value 182.31±0.31 *μ*g/mL, while standard showed the value of 30.12±0.11 *μ*g/mL [53]. In another study also using the DPPH assay,* A. longa* roots had the weakest activity compared to Trolox, among all the plants tested [23]. Furthermore, our study showed similar findings with this work. In ABTS method, we share the same results from a study of five extracts of* A. bracteolate* who found that the methanolic extract had potent antioxidant activity [25]. However, there is variability in the recorded activity of the different extracts; this is certainly related to the difference in the chemical composition of the extracts, especially polyphenols and flavonoids detected by phytochemical analysis in our study. These constituents have different degree of antioxidant activity against different free radicals [55]. The effectiveness of antioxidants may be due to the high amount of the main constituents and also to the presence of other constituents in small quantities or to the synergy between them. Indeed, previous studies reported the presence of chemical compounds such as Aristolactam la (ALIa), Limonen-6-ol, and Maaliol [56–58]. Phenolics are antioxidants with redox properties; the hydroxyl group helps them to work as reducing agents, hydrogen donors and singlet oxygen quenchers [59, 60]. Plant extracts that have high amounts of phenolic compounds do not always translate into high antioxidant capacity; it may be due to the presence of different active compounds, the synergistic effects of these compounds, and also to the position and extent of hydroxylation and conjugation [61]. Moreover, our activity was significantly weak compared to Trolox (*p* <0.05); this result can be interpreted by the fact that the extracts have several compounds, while the concentrations of those who could perform this activity will be really low. It is also noted that the antioxidant effects change depending on the test used. In fact, the antioxidant activity depends on the interactions in the reaction media between the substrate(s) (radicals) and the active molecule(s) that trap them. Other studies have shown that the roots of* A. longa* possess antioxidant activity [23]. Moreover, there is another study that was interested in the aerial part of this plant [52], and we found almost the same results for the DPPH method; IC_50_ values of fruit aqueous extract and fruit methanol extract were found to be 145.15±0.78 *μ*g/mL and 186.21±6.24 *μ*g/mL, respectively. The difference between the results is due, as already mentioned, to the difference of the phytoconstituents, also to the method used, the harvest period, and the studied part (root, leaf, stem…)

### 3.3. *α*-Glucosidase and *β*-Galactosidase Inhibitory Activities

Diabetes is characterized by high blood sugar levels, which can lead to serious complications, so the goal of treating patients with diabetes is to maintain near-normal levels of glycemia control. In modern medicine, there is no treatment or medication to treat diabetes without side effects, which are related to the use of insulin and oral hypoglycemic agents [62]. Medicinal plants with antidiabetic properties may be a useful source for finding safer, cost-effective antidiabetic drugs. In the present research, different extracts of* A. longa* are evaluated for their antidiabetic activity. Two different* in vitro* assays were used to evaluate this activity, *α*-Glucosidase and *β*-Galactosidase uptake assay.

*α*-Glucosidase catalyzes the final step in the digestion of carbohydrates and is located in the brush-border surface membrane of intestinal cells [63]. Its inhibitors can retard the uptake of dietary carbohydrates in the small intestine and reduce postprandial hyperglycemia, which may be a useful mechanism in the preparation of antidiabetic drugs [64]. This is largely used as an effective pharmacological strategy for managing hyperglycemia related to the early stages of type 2 diabetes [65].

On the other hand, *β*-Galactosidase catalyzes the hydrolysis of *β*-Galactosides subtracts to simple carbohydrates in the intestine. Subsequently, the inhibition of this enzyme can lead to the intestinal hydrocarbon reduction and eventually decreases the glucose level.* A. longa* extracts showed inhibitory effects on both enzymes tested as presented in Table 3. The result revealed that the tested extracts inhibited *α*-Glucosidase and *β*-Galactosidase activity concentration dependently (0.5–5 mg/mL). Indeed, at the concentration of 1.5 mg/mL, the ethyl acetate fraction has the highest inhibitory activity against *α*-Glucosidase (76.56±2.54%) and *β*-Galactosidase (12.70±1.27%). The methanolic fraction showed a moderate inhibition of *α*-Glucosidase (21.94±1.34%) and *β*-Galactosidase (2.05±1.22%). However, the aqueous extract inhibited only the enzymatic activity of *β*-Galactosidase (2.20%). These results are consistent with those of Janani and Revathi, [66] who worked on another species of Aristolochiaceae (*A. indica*). Their study revealed that the methanolic extract of whole plant showed *α*-Glucosidase inhibitory activity, which increased with the increasing concentration.

To measure the inhibitory effectiveness of each extract, we used the IC_50_ which represents the concentration of an inhibitor that is required for 50% inhibition of its targeted enzyme. The fractions of ethyl acetate and methanol showed a strong inhibitory capacity against *α*-Glucosidase with IC_50_ values of 1.112±0.026 and 2.378±0.037 mg/mL, respectively. These inhibition values are greater than that obtained by Acarbose (0.199±0.014 mg/mL), used as standard antidiabetic. Similar effects were observed on* A. indica* [67]. Likewise, the *α*-Glucosidase inhibition values by the aqueous extract and aqueous fraction are above 5 mg/mL. For *β*-Galactosidase inhibitory capacity, all extracts showed an IC_50_ value greater than 5 mg/mL. The fact that *α*-Glucosidase and *β*-Galactosidase showed difference is due to structural differences related to the origins of enzymes [68].

The inhibitory effects of* A. longa* extracts against the enzyme *α*-Glucosidase demonstrate their potential abilities to reduce the postprandial increase of blood glucose levels in diabetic patients and their capacities to prevent type 2 diabetes. Hence, it is suggested that the mechanism of antihyperglycemic may be due to the antioxidant activity of this herb. Our finding is in accordance with earlier reports that showed that, in animal models, two of its species, aerial parts of* A. indica *[69] and the* A. ringens* roots extracts [70], showed a reduction in elevated blood glucose level. Moreover, the results are in line with a study performed on 71 herb plants to test their antidiabetic effects that showed that 36 herbs had *α*-Glucosidase inhibition including a species of Aristolochiaceae (*Asarum heterotropoides*) [71]. The differences observed for the inhibitory activity of the enzyme could be explained by the changes in the percentage of inhibition relative to the phytochemical composition of the plant species and also by the sensitivity of enzymes. Consequently, as mentioned above, phytochemical studies on* A. longa* demonstrated its abilities to produce a high amount of phenolic compounds and several flavonoids, including alkaloids [57], saponins, and tannins [72]. The phenolic compounds are known by their capacity to inhibit the activities of carbohydrate-hydrolyzing enzymes because of their ability to bind to proteins [73]. Moreover, the presence of flavonoids, especially in ethyl acetate fraction, may account for the inhibitory activity observed. Indeed, flavonoids have been known to possess high inhibitory potential towards *α*-Glucosidase in both* in vitro* and* in vivo* studies [74] and may prevent the malfunction of pancreatic beta cell due to oxidative stress and can thus reduce the onset of type 2 diabetes [75]. Importantly, some researchers have indicated that there is a positive relationship between total flavonoid and polyphenol content and the ability to inhibit *α*-Glucosidase [76]. The inhibitory effect observed for methanolic fraction of* A. longa* may be associated with the presence of other phytoconstituents like alkaloids, tannins, and saponins [76, 77]. These last have been responsible for suppressing the absorption of liquid and glucose at the brush borders [78]. These compounds, which can also inhibit *α*-Glucosidase, have fewer side effects and are less expensive compared to synthetic pharmacotherapeutics like Acarbose [79], and they perform several other biological activities such as antibacterial, antioxidant, and anticancer [80]. Generally, herbal medicine is based on the therapeutic action of a mixture of different compounds acting often in synergy to exert all their beneficial effects. This suggests that the biologically active compounds present in the extracts studied may act in a synergistic way to exercise their carbohydrate-hydrolyzing enzymes inhibition activities and antioxidant effects. The observed variations in chemical composition of* Aristolochia sp* from different parts of the world are not only due to the type of specie but also different agroclimatic conditions, extraction method, harvest period, and characterization techniques [81, 82] and also to the selected part of plant and polarity of extraction solvents. Concerning plant extracts, other studies have shown that some of them can increase insulin secretion and insulin signaling in adipose and skeletal muscles [83]. In the present study, we have investigated the antidiabetic potential of* A. longa*, which is used in traditional medicine for the treatment of several diseases. This herb was not previously investigated for its* in vitro* antidiabetic activity. The results from this work should be relevant to the human body.

## 4. Conclusion

The aqueous fraction of the* A. longa* roots had the best antioxidant effects against the DPPH and ABTS radicals and a strong ferric reducing power. This suggests that* A. longa* can be used to prevent and control the oxidative stress induced by free radicals. The antidiabetic activity was also investigated, focusing on the inhibitory effects on *α*-Glucosidase and *β*-Galactosidase. Our study is the first to report a potential mode of action of* A. longa* and suggests that the effect of this plant is due to the inhibition of digestive enzymes. On the other hand, the presence of flavonoids and phenols concludes that this herb has multiple biological properties. Other studies must be conducted to isolate the active ingredients of this plant, identify them, and study their bioactivity.

## Figures and Tables

**Table 1:** Total phenolic, flavonoid, and proanthocyanidin content of extracts.

Extracts	Polyphenolic content (mg of GAE/g of extract)	Flavonoid content (mg of RE/g of extract)	Proanthocyanidins content (mg of CE/g of extract)
MF	24.48±1.63^**b**^	7.00±0.61^**b**^	05.78±0.28^**a**^
EAF	32.55±0.43^**c**^	116.86±4.12^**c**^	77.64±1.93^**c**^
AF	28.90±1.36^**b,c**^	07.60±0.11^**b**^	10.79±0.49^**b**^
AE	13.13±0.48^**a**^	03.80±0.32^**a**^	06.87±0.32^**a,b**^

The values are the mean of three determinations ± standard error.

Values in the same column not sharing a common letter (a to c) differ significantly at *p* < 0.05.

MF: methanolic fraction; EAF: ethyl acetate fraction; AF: aqueous fraction; AE: aqueous extract; GAE: gallic acid equivalent; RE: rutin equivalent; CE: catechin equivalent.

**Table 2:** IC_50_ values (*μ*g/mL) of extracts and Trolox on DPPH and ABTS inhibition activity.

Products	DPPH (IC_50_ *μ*g/mL)	ABTS (IC_50_ *μ*g/mL)
MF	199.35±1.25^**b**^	61.58±2.15^**b**^
EAF	220.80±2.40^**b**^	103.62±8.62^**b,c**^
AF	125.40±2.40^**b**^	65.23±2.49^**b**^
AE	354.60±5.20^**c**^	144.40±2.07^**c**^
Trolox	1.79±0.35^**a**^	0.70±0.0^**a**^

The values are the mean of three determinations ± standard error.

Values in the same column not sharing a common letter (a to c) differ significantly at *p*< 0.05.

**Table 3:** *α*-Glucosidase and *β*-Galactosidase enzyme inhibition test results.

Extract/ Standard	*α*-Glucosidase inhibition		*β*-Galactosidase inhibition	
	% of inhibition at 1.5 mg/mL	IC_50_ (mg/mL)	% of inhibition at 1.5 mg/mL	IC_50_ (mg/mL)
Ethyl acetate fraction	76.56±2.54^**b**^	1.112±0.026^**b**^	12.70±1.27^**b**^	>5
Methanolic fraction	21.94±1.34^**a**^	2.378±0.037^**b**^	2.05±1.22^**a**^	>5
Aqueous fraction	na	>5	na	>5
Aqueous extract	na	>5	2.20±0.13^**a**^	>5
Acarbose	96.78±0.03^**c**^	0.199±0.014^**a**^	----	----
Quercetin	----	----	92.62±0.14^**c**^	0.247±0.006

na: nonactive; mean ± SD (n=3).

Values in the same column not sharing a common letter (a to c) differ significantly at *p*< 0.05.

## Data Availability

The data used to support the findings of this study are included within the article.
